# Development and psychometric evaluation of the healthy lifestyle questionnaire for elderly (heal)

**DOI:** 10.1186/s12955-020-01529-3

**Published:** 2020-08-12

**Authors:** Razieh Bandari, Farahnaz Mohammadi Shahboulaghi, Ali Montazeri

**Affiliations:** 1 Social Determinants of Health Research Center, Semnan University of Medical Sciences, Semnan, Iran; 2 Full Professor of Iranian Research Center on Aging, Nursing Department, University of Social Welfare and Rehabilitation Sciences, Tehran, Iran; 3 Mental Health Research Group, Health Metrics Research Centre, Iranian Institute for Health Sciences Research, ACECR, Tehran, Iran; 4 Faculty of Humanity Sciences, University of Science & Culture, ACECR, Tehran, Iran

**Keywords:** Elderly, Healthy lifestyle, Psychometric, Development, Validity, Reliability

## Abstract

**Background:**

The present study was conducted to design and evaluate the psychometric properties of a questionnaire for assessing the healthy lifestyle among older adults in Iran.

**Methods:**

First, items were generated based on a qualitative study, the literature review, and with help received from experts in gerontology and questionnaire design. Then, content validity was carried out. Accordingly, a cross sectional study was conducted to perform factor analysis and known groups comparison in order to examine the construct validity. Internal consistency was measured by the Cronbach’s alpha coefficient and the stability of the questionnaire was evaluated by estimating interclass correlation coefficient (ICC).

**Results:**

In total 68 items was generated. Following development process 22 items were removed and a provisional version of the questionnaire with 46 items was subjected to psychometric evaluation. At this stage a sample of 390 elderly people attending the community centers in Tehran, Iran were entered into the study and completed the questionnaire. Most elderly were female (52.8%) and the mean age of participants was 67.97 (SD ± 7.77) years. After performing factor analysis, overall 10 items were removed due to low loading and the questionnaire was reduced to 35 items tapping into eight factors, which explained a total of 57.1% of the variance. In addition, the results obtained from known groups comparison indicated that the questionnaire well differentiated among participants who were differed in self-reported health condition. The Cronbach’s alpha coefficient showed excellent internal consistency (alpha = 0.89). The intraclass correlation coefficient also indicated a good stability for the questionnaire (ICC = 0.94).

**Conclusion:**

The healthy lifestyle questionnaire for elderly (Heal) can be used as a simple and an easy-to-use valid and reliable measure in determining healthy life style and the frequency of health-oriented activities among older adults.

## Background

Since the beginning of the twenty-first century, improved living conditions and life expectancy have resulted in an increase in the population of older adults worldwide. For instance, it has been reported that between 2015 and 2030 the older population in lower-middle income countries will increase by 66% and that in low-income countries will grow up by 63% [1]. However, as far as it relates to Iran, the official statistics indicates that the population of older adults is increasing steadily. At present about 10% of the population of Iran are aged 60 and over [2, 3].

Owing to the accelerated phenomenon of population aging in Iran as in other countries, demographic conditions are changing and moving toward aging and its resultant consequences [4]. These changes will most likely lead to important economic, health, and social challenges, and a growing prevalence of chronic conditions will increase the need for health improvement interventions by healthcare providers as well as family members and communities. It must be noted that modification and improvement of lifestyle are important prerequisites for maintaining good health [5] Given the growth in chronic conditions and, based on the health promotion approaches, people must be empowered to accept responsibility for their own health and adopt a healthy lifestyle [6].

A healthy lifestyle with the multidimensional nature is crucial, as it can influence the quality of life, illness management, and prevent diseases [7]. A healthy lifestyle means having a balanced life in which an individual consciously makes healthy choices and takes special measures such as following a healthy diet, striking a balance among sleep, activity, and exercise, controlling weight and stress, abstaining from smoking and consuming alcohol, and getting oneself immunized against diseases [8]. However, though lifestyle is formed by personal choices and identities, it cannot be analyzed in isolation of its social and cultural contexts. It means the personal, biological and psychological characteristics of the individual, family, friends, and social community affect the individual’s daily life and lifestyle [9].

As there seems to be a triangular relationship between aging, chronic conditions, and healthy life style, the assessment of attitudes and behaviors of the elderly toward a certain lifestyle can offer information to healthcare providers to evaluate elders’ way of living and accurately design appropriate preventive interventions, enhance the capabilities of the elderly, and modify their lifestyle in an acceptable manner if necessary.

Assessment of the lifestyle of older people requires accurate tools for measuring health-related behaviors. Although there are numerous instruments for the measurement of lifestyle in Western communities, differences in social and cultural contexts, especially the ethnic backgrounds [9] and educational levels of older adults [10], might cause some limitations to their use in other communities such as Iran, where more than two-thirds of the elderly people are either illiterate or less educated [11]. Additionally some existing instruments only measure special lifestyle aspects for example, stress management, nutrition or exercise and do not have comprehensive approach to measuring healthy lifestyles.

Among existing instruments, the Health Enhancement Lifestyle Profile (HELP II), as a common multidimensional tool, has been applied in most lifestyle studies [12, 13]. The Iranian version of this questionnaire has been developed and mainly is used for young people [5, 8, 14–17].

Considering the limitations of using the existing tools in older adults, Eshaghi et al., in 2010, designed a Healthy Lifestyle Assessment Questionnaire for Iranian older adults. This questionnaire was designed based on a review of available tools, relevant texts, and interviews with Iranian elderly population. It includes 46 Likert type questions, which are designed to assess physical activity, sport and recreation, healthy eating, stress management, and social and interpersonal relationships in older adults [18]. This questionnaire is long and the response categories are very diverse and sometimes could be difficult to understand and respond to its items by elders.

According to Haywood et al., despite the presence of numerous instruments, it still is necessary to design and develop appropriate specific tools for screening and monitoring the health status and lifestyle of the elderly population. They argue that such tools must be simple, comprehensible, and easily applicable especially in clinical settings. In addition, as they should be more acceptable for and focused on the status of the elderly, they should be able to monitor and evaluate the changes in the elderly health status more precisely [18]. Spencer et al. believe that the evaluation of the health status of the elderly by the use of short questions and without clinical computation will facilitate the assessment of their health status and provide more valid and reliable data not only for healthcare providers but also for researchers [19].

Indeed, the nature of the aging process, some abilities, such as the loss of hearing, vision, concentration, and memory decline in older adults, can lead to problems in effective communication. Such problems make it difficult to use instruments with long and unclear statements for questioning older people. Given the lack of an appropriate tool to study the healthy lifestyle of the elderly population, it seems necessary to design instruments of acceptable validity and reliability that would be consistent with norms and social contexts, and be simple and easily applicable, especially to illiterate elders. Accordingly, the present study was designed to develop a questionnaire to study healthy lifestyles among older adults and evaluate its validity and reliability.

## Methods

This was a methodological study. The study was carried out in four phases. These are described as follows:

### Item generation

In order to generate items for the questionnaire a review of the literature, and a qualitative study was conducted to form the concept of a healthy lifestyle for elderly.

1. Review of the literature: A purposeful electronic search was carried out using PubMed’ search engine and the ‘Science Direct’ portal. Articles with keywords including psychometric evaluation, measurement, healthy lifestyle, elderly, aging, old, and health behaviors were retrieved in order to develop the concept and to generate items for the questionnaire.

2. Qualitative study: Semi-structured interviews were conducted to elucidate determinates of healthy life style among elderly as perceived by experts and elderly people. In doing so eleven experts (nurses, physiotherapists, occupational therapists, general practitioners, psychiatrists, psychologists and gerontologists) and sixteen older adults (8 men and 8 women) aged 60–75 shared and discussed their experiences on strategies to maintain and improve health among elderly seniors. Purposive sampling with maximum variance (age, marital status, education, socio-economic backgrounds and living conditions) was used to choose elderly participants from members of elderly health clubs affiliated to the Tehran municipality livening in different districts of the city. Each interview lasted for about 45 to 70 min and was recorded and immediately transcribed verbatim. The transcribed text was read several times, and the codes were extracted [20].

3. Item pool: A total of 68 items were generated from the above-mentioned procedures. Of these, 21 items were derived from the literature review, 20 items derived from expert interviews and 27 items extracted from interviews with older people.

### Item reduction

At this stage the research team evaluated items and merged the expressions with overlaps, and excluded those items that seemed irrelevant to healthy life style. Thus the initial version of the questionnaire with 55 items was subjected to the further item reduction process.

Content validity: The objective of content validity was to determine the degree to which the questionnaire was able to measure the intended concept under evaluation [21]. To do so, we used both qualitative and quantitative methods and asked 15 psychometrics, gerontology, public health and geriatrics to evaluate the items. First the experts were asked to assess the questionnaire based on grammar, wording, the right placement of items, as well as the right scoring, and provide the necessary feedback. Then, the quantitative content validity was performed by estimating the content validity ratio (CVR) and the content validity index (CVI). Regarding the CVR, the experts were asked about the necessity of each item, and a CVR value of > 0.45 was considered acceptable [22]. Regarding the CVI, the criteria of relevance, clarity, and simplicity of the items were assessed and a CVI value of > 0.79 was considered acceptable. As a result at this stage 9 items were removed and the provisional version of the questionnaire with 46 items made ready for pre-test evaluation without any prior assumptions or considering a theoretical model.

### Pre-test

To determine initial internal consistency, the Cronbach’s alpha coefficient of the elderly healthy lifestyle questionnaires was calculated. As such a pilot sample of 60 elderly people completed the questionnaire and the Cronbach’s alpha coefficient was found to be 0.90. Then another sample of twelve elderly people completed the questionnaire. They were asked to comment on comprehensibility, relevance, and ambiguity. They all received the questionnaire well and indicated that it was easy to understand and they did not have any difficulties to complete the questionnaire.

### Psychometric evaluation

1. Design and participants: A cross sectional study was conducted in Tehran, Iran in order to evaluate the psychometric properties of the Healthy Life Style for Elderly (Heal) questionnaire. Using the random sampling method, a total of 390 eligible elderly people were selected from members of the elderly health clubs affiliated to the Tehran municipality. For this purpose, Tehran was divided into five regions: north, south, east, west, and the central part. Then, from each region two clubs were randomly selected. Again, random sampling was applied to select the elderly from members of each of the clubs, based on the table of random numbers. The inclusion criteria for including samples in the study were as follows: being aged 60 and older, no hearing and vision deficit as per self-reports, no cognitive decline (obtaining a score of seven or higher in the Iranian Version of the Abbreviated Mental Test) [23], and willingness to participate in the study. The sample size was estimated based on the required samples that needed for the factor analysis [24].

2. Construct validity: The following procedures were applied to examine the construct validity.

2.1 Structural validity: In order to test and determine the structural validity of the questionnaire, exploratory factor analysis was performed.

2.2 Known-groups comparison: Known-groups comparison was used to determine the extent to which the questionnaire was able to distinguish among different subgroups of particpants who differed in health status. Health status was measured using a self-reported single item that rated on a five point likert scale.

3 Reliability of the questionnaire: Reliability was measured by examining internal consistency and test-retest reliability. For the purpose of test-retest reliability a subsample of 30 elderly completed the questionaire twcie with a two-weeks interval. It was insured that in the interim period between the two administrations the samples’ health-related lifestyle and behaviors did not change.

### Statistical analysis

The Shapiro-Wilk test was performed to assess data normality. Descriptive statistics were used to create an overview of the sample characteristics. Psychometric evaluation was performed several statistical procedures. The maximum likelihood approach, and varimax rotation was used to examine the structural validity. The adequacy of sample size was assessed using KMO statistic, which should be at least 0.06, and by Bartlett’s test of sphericity, which should be statistically significant [25, 26]. Items were considered for deletion if loadings on any of the components were less than 0.4 [27, 28]. Using the one-way analysis of variance (ANOVA) with Bonferroni method for post hoc the life style score among different sub-groups of sample was compared to assess known-groups validity. Reliability was measured by examining internal consistency and test-retest reliability. Internal consistency was assessed using the Cronbach’s alpha coefficients (α) and values of 0.70 or above were considered satisfactory [3]. Test-retest reliability was estimated by calculating the intraclass correlation coefficient-ICC (two-way mixed effects model, single measure) and values of 0.75 were thought acceptable [3].

## Results

### Questionnaire development

The healthy lifestyle questionnaire for elderly (Heal) was developed based on a robust methodological procedure. The results obtained from pre-test showed that the questionnaire received well and almost all participants in this stage (*n* = 12) indicated that the questionnaire was easy to understand, and they could rate the items easily. A few participants suggested it would be better to print the questionnaire in a larger font. However, after performing factor analysis the final questionnaire contained 35 items. Each item is rated on a 5-point Likert scale (always, most of the time, sometimes, rarely, never). The row score for the questionnaire ranges from 35 to 175, which with a simple linear transformation could be converted to 0 to 100, where the higher scores indicate better healthy life style.

### The main study: participants

In all 390 elderly took part in the study. Of these 205 (52.4%) were female, 68.0% (*n* = 365) were married, and 58% were retired. Most participants reported that they are living with family (36.4%) and indicated themselves as having intermediate economic status (41.6%). The characteristics of the participants are shown in Table 1.

**Table 1:** The characteristics of study participants (*n* = 390)

	Number (%)
**Gender**	
Male	185 (47.4)
Female	205 (52.6)
**Age group (years)**	
60–70	271 (69.4)
71–80	103 (26.4)
80<	16 (4.2)
**Educational**	
Illiterate	32 (8.2)
Primary	154 (39.4)
Secondary	116 (29.8)
Higher	88 (22.6)
**Marital status**	
Married	265 (68.0)
Single	10 (2.6)
Widowed	108 (27.6)
Divorced	7 (1.8))
**Employment status**	
Housewife	137 (35.0)
Employed	27 (7.0)
Retired	226 (58.0)
**Number of children**	
0	19 (4.8)
1–3	176 (45.2)
4–6	174 (44.6)
> 7	21 (5.4)
**Living condition**	
Alone	80 (20.6)
With spouse	118 (30.2)
With children	46 (11.8)
With family	142 (36.4)
Others	4 (1.0)
**Economic status**	
Poor	82 (27.0)
Intermediate	162 (41.6)
Good	146 (31.4)
**Housing**	
Owner	310 (79.6)
Tenant	71 (18.20
Children’s home	5 (1.2)
Relative’s home	4 (1.0)
**Health status**	
Poor/Very poor	153 (38.2)
Fair	174 (43.2)
Very good/Good	63 (15.6)
**History of the disease**	
Yes	149 (49.8)
No	241 (50.2)

**Fig. 1:**
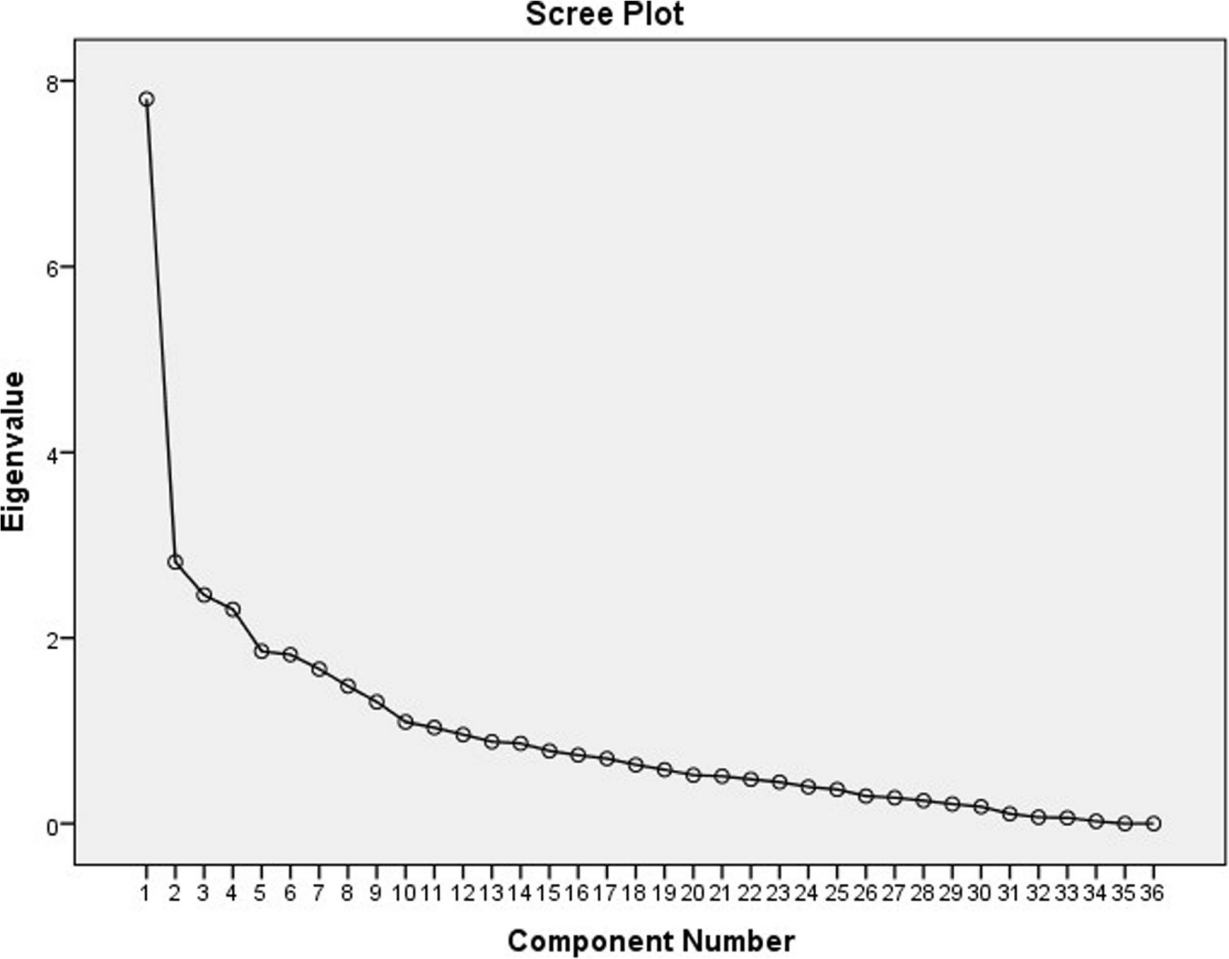
A scree plot illustrating the factor loading of the healthy lifestyle questionnaire for elderly (Heal)

### Structural validity

The KMO value was found to be 0.85. In addition, the Bartlett’s test for Sphericity had a value of 4425.38, and was significant at 0.0001 level. The latent factors were extracted using the maximum likelihood estimation and the varimax rotation. Based on the results obtained from factor analysis, since the factor loading of 10 items was less than 0.4, thus these were removed and the questionnaire was reduced to 35 items. However, eight factors were extracted, based on eigenvalues above 1 and the scree plot (Fig. 1). As shown in Table 2, the factors jointly explained 57.1% of the variance observed.

**Table 2:** Exploratory factor analysis of the healthy lifestyle questionnaire for elderly (Heal)

	F1	F2	F3	F4	F5	F6	F7	F8
1. How often do you spent time on personal hygiene activities such as brushing teeth, washing hands and face, trimming nail, etc.?	**0.72**	0.32	0.22	0.20	0.41	0.35	0.49	0.14
2. How often do you take a bath or take a shower?	**0.78**	0.16	0.12	0.25	0.34	0.49	0.21	0.49
3. How often do you buy the things you need, independently?	0.34	**0.51**	0.19	0.32	0.04	0.03	0.21	0.02
4. How often do you cook or prepare a food to eat (Or help someone else to do this for you)?	0.03	**0.93**	0.21	0.10	0.03	0.01	0.06	0.02
5. How often do you clean your home or give it a tidy?	0.16	**0.91**	0.28	0.16	0.14	0.07	0.06	0.06
6. How often do you walk at least for 20–30 min per day?	0.06	0.03	**0.60**	0.21	0.20	0.02	0.05	0.06
7. How often do you exercise or play sports other than walking?	0.21	0.09	**0.74**	0.32	0.20	0.01	0.09	0.012
9. How often do you eat white meat (poultry, fish, chicken, etc.)?	0.03	0.09	0.11	**0.68**	0.16	0.02	0.08	0.10
10. How often do you use liquid oils for cooking?	0.06	0.10	0.12	**0.51**	0.16	0.02	0.12	0.11
12. How often do you eat raw vegetables (vegetables, salad, lettuce, etc.) or cooked vegetables (squash, eggplant, etc.)?	0.20	0.20	0.01	**0.58**	0.02	0.17	0.21	0.12
13. How often do you eat fresh fruits?	0.12	0.24	0.30	**0.66**	0.27	0.15	0.18	0.16
14. How often do you consume foods containing calcium (Like milk, cheese, yogurt, orange, cabbage, etc.)?	0.02	0.18	0.21	**0.69**	0.14	0.11	0.17	0.12
16. How often do you add salt to your food at the table?	0.01	0.13	0.14	**0.66**	0.24	0.10	0.20	0.12
19. How often do you eat complete breads with whole grain?	0.34	0.02	0.02	**0.48**	0.05	0.08	0.19	0.12
20. How often do you drink at least eight glasses of water a day?	0.22	0.31	0.08	**0.62**	0.17	0.04	0.11	0.13
21. How often do you feel satisfied with your night’s sleep?	0.09	0.24	0.03	0.04	**0.69**	0.02	0.08	0.12
22.How often do you go to bed at night on time?	0.32	0.24	0.08	0.03	**0.62**	0.04	0.08	0.11
24. How often can you control your anger and anxiety?	0.19	0.03	0.09	0.14	.**0.64**	0.09	0.11	0.01
25. How often do you try to get rid of sorrow and depression?	0.09	0.24	0.16	0.20	**0.72**	0.02	0.16	0.14
40.How often do you entertain yourself (for instance through gardening, making handcrafts, studying newspaper or book, playing chess, weaving, watching television, listening to radio, etc.)?	0.05	0.11	0.05	0.20	**0.77**	0.04	0.11	0.02
26. How often do you avoid tasks that are potentially harmful for you (lifting heavy objects, going up and down stairs too frequently, going up a ladder or a seat, etc.)?	0.09	0.10	0.21	0.16	0.21	**0.42**	0.30	0.08
28. How often do you take the medication prescribed by your doctor on a regular basis?	0.18	0.15	0.14	0.16	0.17	**0.49**	0.13	0.01
31. How careful are you to maintain a healthy weight?	0.03	0.14	0.06	0.02	0.13	**0.66**	0.17	0.22
32. How often do you follow health and hygiene instructions (so that you don’t get sick)?	0.09	0.06	0.12	0.04	0.15	**0.70**	0.17	0.22
33. How often do you visit a doctor for check-ups?	0.30	0.12	0.07	0.14	0.08	**0.66**	0.10	0.06
34. How often do you monitor your level of blood sugar, fat, or blood pressure?	0.33	0.37	0.03	0.24	0.27	**0.71**	0.43	0.02
35. How often do you care about safety standards to prevent falls, burns, injuries, slipping in the shower and toilet, etc.?	0.13	0.29	0.11	0.05	0.24	**0.52**	0.06	0.06
39.How often do you smoke cigarettes or other tobacco products (tobacco, pipe, hookah, etc.)?	0.19	0.10	0.07	0.17	0.11	**0.58**	0.23	0.30
36.How often do you meet your friends and family?	0.16	0.19	0.09	0.07	0.10	0.21	**0.80**	0.10
37.How often do you talk on the phone with relatives and friends?	0.25	0.13	0.16	0.18	0.08	0.26	**0.85**	0.21
41.How often do you perform religious activities (like pray, reading prayer and religious books, visiting religious places, shrines, mosque, church, etc.)?	0.17	0.07	0.10	0.11	0.09	0.07	0.16	**0.59**
43. What times do you feel satisfied with your life?	0.16	0.13	0.13	0.06	0.04	0.05	0.28	**0.67**
44. How often do you try to achieve your dreams and goals?	0.15	0.23	0.02	0.27	0.07	0.17	0.04	**0.60**
45. What times do you rely on God (or super power)?	0.13	0.11	0.09	0.01	0.06	0.23	0.10	**0.66**
46. What times do you expect a good future?	0.08	0.01	0.04	0.09	0.08	0.03	0.12	**0.79**
8: How often do you eat red meat (beef, mutton, lamb ...)?	0.04	0.10	0.09	0.09	0.15	0.06	0.16	0.29
11: How often do you use solid, animal, butter or tail oil to cook food?	0.05	0.13	0.05	0.03	0.19	0.01	0.02	0.27
15: How often do you eat fatty foods (butter, cream, offal, sandwiches, pizza ......)?	0.04	0.01	0.13	0.10	0.18	0.06	0.12	0.01
17: How often do you eat dried fruits and nuts (almond and pistachio kernels, hazelnuts, seeds ......)?	0.33	0.02	0.08	0.04	0.05	0.11	0.01	0.09
18: How often do you consume sweets (sweeter, creamy, dry, candy, candy, etc. sweet syrup ...)?	0.16	0.08	0.06	0.06	0.10	0.04	0.08	0.09
23: How often do you feel tired?	0.04	0.09		0.21	0.23	0.09	0.04	0.03
27: How often do you get enough rest?	0.01	0.05	0.25	0.10	0.12	0.11	0.02	0.10
29: How often do you take painkillers (acetaminophen, ibuprofen, gelofen, etc.) for body aches?	0.25	0.02	0.02	0.03	0.05	0.18	0.04	0.04
30: How often do you take supplements such as vitamins, iron pills, calcium, fish oil, etc.?	0.11	0.06	0.15	0.17	0.15	0.01	0.09	0.29
38: How often do you participate in group and volunteer work in your neighborhood, such as: religious delegations, public meetings of buildings or apartments, solving neighborhood problems, helping neighbors, and so on?	0.05	0.15	0.07	0.17	0.12	0.08	0.01	0.27
42: How often have you used traditional therapies such as herbal decoctions (oxtongue, cupping, herbal extracts (mint extract, pussy extract .....), massage, acupuncture ...?	0.07	0.02	0.04	0.13	0.06	0.05	0.04	0.01
*Eigenevalue*	9.49	8.43	8.33	6.78	6.59	6.19	6.19	5.14
*% variance*	8.56	2.47	1.49	1.80	1.75	1.47	1.29	1.27

**F1:** Personal health and hygiene, **F2:** Performing life tasks independently, **F3:** Exercise, **F4:** Nutrition, **F5:** Mental Health**, F6:** Safety and health advice, **F7:** Social & family relationships, **F8:** Spirituality and religious practice

### Known groups comparison

The results obtained from one-way analysis of variance showed that the Heal was able significantly differentiate among subgroups of elders who were differed in health status. Those elderly who reported as having very good or good health significantly scored higher on all subscales of the healthy lifestyle questionnaire (*P* < 0.005). The results are presented in Table 3.

**Table 3:** Comparison of the mean row scores of healthy lifestyle questionnaire for elderly by health status (the known-groups comparison)

	Very good/Good (***n*** = 153)	Fair (***n*** = 174)	Poor/Very poor (***n*** = 63)	***P***
	Mean (SD)	Mean (SD)	Mean (SD)	
Personal health and hygiene	8.96 (0.82)	8.88 (0.78)	8.78 (0.96)	0.05
Performing life tasks independently	10.75 (2.92)	10.58 (3.01)	9.79 (3.04)	0.01
Sport	6.92 (1.92)	6.40 (1.90)	5.66 (1.94)	.0001
Nutrition	30.09 (4.10)	29.38 (3.48)	27.76 (4.27)	0.001
Mental Health	18.85 (3.16)	18.63 (3.12)	18.01 (3.29)	0.03
Observing safety and health advice	31.30 (4.06)	31.09 (3.40)	30.26 (3.81)	0.03
Social and family relations	8.67 (1.33)	8.32 (1.32)	7.69 (1.47)	0.0001
Spiritual and religious activities	21.09 (4.11)	20.40 (3.52)	17.46 (3.70)	0.0001
Total scale	133.34 (13.99)	133.73 (11.86)	125.46 (13.14)	0.0001

### Reliability

The Cronbach’s alpha coefficient was calculated for the whole questionnaire as well as for each factor. The Cronbach’s alpha coefficients ranged from 0.70 to 0.97; well above acceptable threshold. The alpha coefficient for the scale as a whole was 0.89. The ICC was 0.94; reflecting a good test-retest reliability (Table 4).

**Table 4:** The Cronbach’s alpha and the Intraclass Correlation Coefficients (ICC) for healthy lifestyle questionnaire for elderly (Heal)

	Number of items	Cronbach’s alpha	Interclass correlation coefficient (95% CI)
**Personal health and hygiene**	2	0.87	0.90 (0.90–0.93)
**Performing life tasks independently**	3	0.97	0.98 (0.99–0.99)
**Sport**	2	0.95	0.98 (0.99–0.99)
**Nutrition**	8	0.70	0.83 (0.76–0.94)
**Mental Health, sleep and rest**	5	0.73	0.99 (0.99–0.99)
**Observing safety and health advice**	8	0.71	0.91 (0.84–0.95)
**Social and family relations**	2	0.78	0.80 (0.68–0.91)
**Spiritual and religious activities**	5	0.93	0.97 (0.91–0.92)
**Total**	35	0.89	0.94 (0.88–0.97)

## Discussion

Healthy lifestyle is a multidimensional concept that has been discussed in the literature for many years and several studies have been carried out to investigate how to measure it. The purpose of this study was to design and psychometrically appraise an instrument to measure the healthy lifestyle among elderly papulation. It is generally believed that a healthy lifestyle is a way of life that reduces the risk of diseases and reduces premature deaths. In fact, the concept could be determined by people’s patterns of behavior and as a characteristic of a balanced life [29]. On the other hand, it is also could be affected by social factors. Thus, it can say that, lifestyle is a complex interaction between different physical and psychological factors. To achieve this the initial items for the current questionnaire were originated and developed based on data from a qualitative study and review of the literature.

The validity of the questionnaire was assessed using factor analysis. Eight domains were identified. The context of these eight domains (personal health and hygiene, performing life tasks independently, sport, nutrition, mental health, observing safety and health advice, social and family relations, spiritual and religious activities) was consistent with the theoretical foundation and structure defined for healthy lifestyle for elderly. These eight domains explained 57.1% of the cumulative variance observed in the results. However, one should note that the questionnaire might not be relevant to clinical settings although it might help clinicians to assess the extent to which elderly populations adhere to clinical and medical advice and treatment.

The results from the psychometric assessment indicated that the questionnaire had a suitable reliability. The Cronbach’s alpha for the subscales ranged from 0.70 to 0.97, showing a good internal consistency for the questionnaire. Test-retest assessment revealed that the questionnaire was reliable and could be used in different time frames.

Considering the great validity and reliability of the healthy lifestyle questionnaire, as well as its advantages such as the relatively low number of questions, simple and easy implementation, and its particular design for assessing the healthy lifestyle among various groups of elderly, it is an appropriate questionnaire for assessing healthy lifestyle.

While answering the items of a questionnaire, people must utilize their cognitive abilities to assess the integrity and legitimacy of the questions, recall information from memory, evaluate the relationship between recalled information and the questions in the questionnaire, and transfer the response [30]. Since the elderly face a normal decline of cognitive capabilities, it is recommended that when designing a questionnaire, the type of questions and the likely problems that may emerge during the use of the questionnaire should be considered. There are several factors that may cause measurement errors in the elderly such as hearing and vision problems, anxiety, being affected by multiple chronic illnesses, and differences in the format of the questions in a questionnaire [10]. The existing evidence highlights the need for being precise and simple in questionnaire application for the elderly [19]. Taking into account the above-mentioned facts, we believe the application of our questionnaire even for the illiterate and less educated elderly will be easy. It is relatively short, easy-to-use, and measures different dimensions of healthy life style and takes about 15 to 20 min to be completed.

### Strengths and limitations

Among the strengths of the present study were a relatively large sample, random sampling, and performing expletory factor analysis. Some limitations include lack of purposeful target population use for the content validity (CVI and CVR). In addition, we did not perform convergent/divergent validity. Perhaps performing such analyses would lead to the stronger psychometric properties of the questionnaire. The future studies also might benefit from performing a confirmatory factor analysis to see if could verify the current structure of the questionnaire.

## Conclusion

The findings suggest that the healthy life style questionnaire for elderly (Heal) is a valid measure. However, to confirm its firm validity, further evaluations are recommended.

## Data Availability

The datasets are available from the corresponding authors on request.

## Abbreviations

CVI: Content Validity Index
CVR: Content Validity Ratio
EFA: Exploratory Factor Analysis
KMO: Kaiser–Meyer–Olkin

## References

[1] United nations, Department of Economic and Social Affairs, Population Division (2015). World Population Aging 2015 (ST/ESA/SER.A/390).

[2] Bandari R, Heravi-Karimooi M, Miremadi M, Mohebbi L, Montazeri A (2019). The Iranian version of geriatric anxiety inventory (GAI-P): a validation study. Health Qual Life Outcomes.

[3] Miremadi M, Bandari R, Heravi-Karimooi M, Rejeh N, Sharif Nia H, Montazeri A (2020). The Persian short form aging perceptions questionnaire (APQ-P): a validation study. Health Qual Life Outcomes.

[4] Tanjani PT, Motlagh ME, Nazar MM, Najafi F (2015). The health status of the elderly population of Iran in 2012. Arch Gerontol Geriatr.

[5] Hosseini M, Yaghmaei F, Hosseinzade S, Alavi Majd H, Sarbakhsh P, Tavousi M (2012). Psychometric evaluation of the “health promoting life style profile 2”. Payesh..

[6] Fotoukian Z, Shahboulaghi FM, Khoshknab MF, Mohammadi E (2014). Concept analysis of empowerment in old people with chronic diseases using a hybrid model. Asian Nurs Res (Korean Soc Nurs Sci).

[7] Heravi-Karimooi M, Rejeh N, Garshasbi A, Montazeri A, Bandari R (2018). Psychometric properties of the Persian version of the quality of life in early old age (CASP-19). Iran J Psychiatry Behav Sci.

[8] Mohamadian H, Eftekhar Ardebili H, Taghdisi MH, Mousavi GA, Sabahi-Bidgoli M (2013). Psychometric properties of the health-promoting lifestyle profile (HPLP II) in a sample of Iranian adolescents. Payesh..

[9] Ghanei M, Ahmady K, Babaei M, Tavana AM, Bahadori M, Ebadi A (2016). Knowledge of healthy lifestyle in Iran: a systematic review. Electron Physician.

[10] Burnside I, Preski S, Hertz JE (1998). Research instrumentation and elderly subjects. Image J Nurs Sch.

[11] Khosravi A, Alizadeh M, Torkashvand M, Aghaei N (2014). Population ageing in IR Iran: UNFPA.

[12] Hwang JE (2010). Promoting healthy lifestyles with aging: development and validation of the health enhancement lifestyle profile (HELP) using the Rasch measurement model. Am J Occup Ther.

[13] Hwang JE (2010). Reliability and validity of the health enhancement lifestyle profile (HELP). Am Occup Ther Foundation.

[14] Maheri AB, Bahrami MN, Sadeghi R (2013). The situation of health-promoting lifestyle among the students living in dormitories of Tehran University of Medical Sciences. Iran J Health Development.

[15] Safabakhsh L, Nazemzade M (2013). The effect of health promotion education on high school students’ lifestyle. Iranian J Med Educ.

[16] Tol A, Tavassoli E, Shariferad GR, Shojaezadeh D. The Relation between Health-promoting lifestyle and quality of ;ife in undergraduate students at school of health, Isfahan University of Medical Sciences, Iran. Health System Res. 2011;7(4):1–6. 10.4103/2277-9531.108006 PMC3778574 24083261

[17] Eshaghi SA, Farajzadegan Z, Babak A. Healty lifestyle assessment questionnaire in elderly: translation, reliability and validity. Payesh. 2010;9(1):91-9.

[18] Haywood KL, Garratt AM, Fitzpatrick R. Older people specific health status and quality of life: a structured review of self-assessed instruments. J Eval Clin Pract. 2005;11(4):315-27. 10.1111/j.1365-2753.2005.00538.x 16011644

[19] Spencer CA, Jamrozik K, Norman PE, Lawrence-Brown M. A simple lifestyle score predicts survival in healthy elderly men. Prev Med. 2005;40(6):712-7. 10.1016/j.ypmed.2004.09.012 15850869

[20] Graneheim UH, Lundman B. Qualitative content analysis in nursing research: concepts, procedures and measures to achieve trustworthiness. Nurse Educ Today. 2004;24(2):105-12. 10.1016/j.nedt.2003.10.001 14769454

[21] Firouzbakht M, Tirgar A, Ebadi A, Sharif Nia H, Oksanen T, Kouvonen A, et al. Psychometric Properties of Persian Version of the Short-Form Workplace Social Capital Questionnaire for Female Health Workers. Int J Occup Environ Med. 2018;9(4):184-93. 10.15171/ijoem.2018.1264 PMC6466993 30325359

[22] Azizi N, Karimy M, Abedini R, Armoon B, Montazeri A. Development and Validation of the Health Literacy Scale for Workers. Int J Occup Environ Med. 2019;10(1):30-9. 10.15171/ijoem.2019.1498 PMC6522212 30685775

[23] Foroughan M, Wahlund LO, Jafari Z, Rahgozar M, Farahani IG, Rashedi V. Validity and reliability of Abbreviated Mental Test Score (AMTS) among older Iranian. Psychogeriatrics. 2017;17(6):460-5. 10.1111/psyg.12276 28589659

[24] Polit DF, Yang F. Measurement and the Measurement of Change: A Primer for the Health Professions. Philadelphia: Lippincott Williams & Wilkins; 2015.

[25] Brown T, Onsman A. Exploratory factor analysis: A five-step guide for novices. Australasian Journal of Paramedicine. 2010;8:1-13.

[26] Pallant J. SPSS survival manual. A step by step guide to data analysis using IBM SPSS. 5th ed. Maidenhead: Open University Press/McGraw-Hill; 2016.

[27] Hahs-Vaughn DL. Applied Multivariate Statistical Concepts. Oxfordshire United Kingdom: Taylor & Francis; 2016.

[28] DeVon HA, Block ME, Moyle‐Wright P, Ernst DM, Hayden SJ, Lazzara DJ, et al. A psychometric toolbox for testing validity and reliability. Journal of Nursing Scholarship. 2007;39(2):155-64. 10.1111/j.1547-5069.2007.00161.x 17535316

[29] Farhud DD. Impact of Lifestyle on Health. Iranian Journal of Public Health. 2015;44(11):1442-4. PMC4703222 26744700

[30] Mohammadi F, Eftekhari MB, Dejman M, Forouzan AS, Mirabzadeh A. Seeking comfort: women mental health process in I. R. Iran: a grounded theory study. Int J Prev Med. 2014;5(2):217-23. PMC3950746 24627750

